# Supplementary material for: Structural basis for receptor recognition of pollen tube attraction peptides
Source: Nat Commun. 2017 Nov 6;8:1331. doi: 10.1038/s41467-017-01323-8 (PMC5673899; doi:10.1038/s41467-017-01323-8)
Supplement: Supplementary file 1 — Supplementary Information [file 41467_2017_1323_MOESM1_ESM.pdf]

Supplementary Information

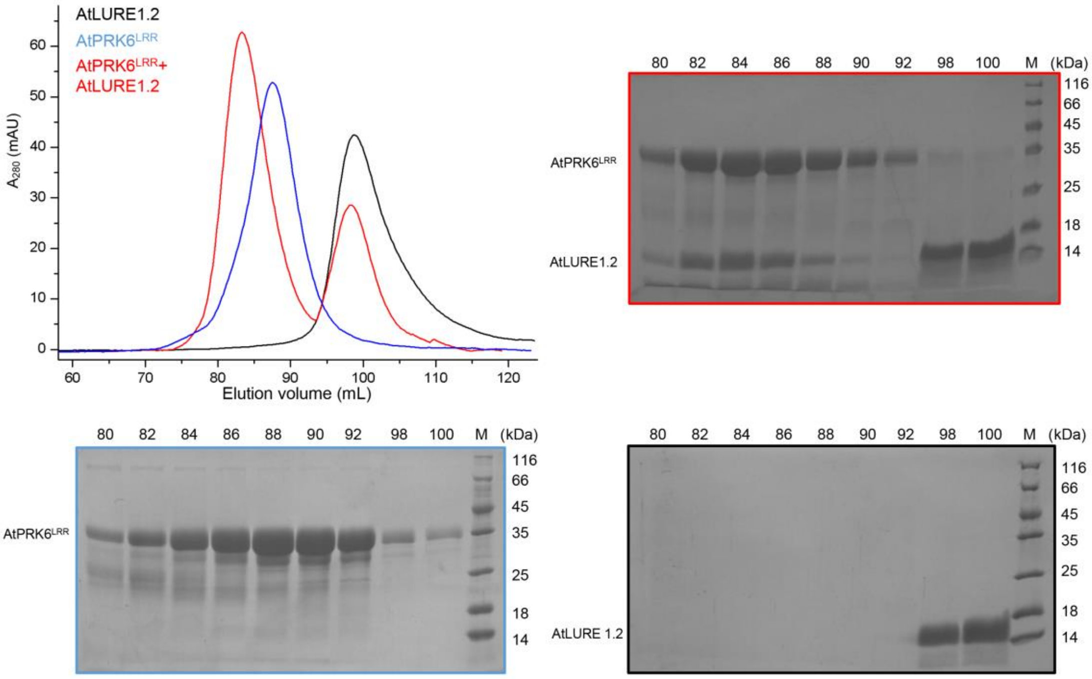

Supplementary Figure 1: Full blot of SDS-PAGE panels in Figure 1b.
Gel-filtration profiles of AtPRK6^{LRR}, AtLURE1.2 and AtPRK6LRR-AtLURE1.2 complex. A_{280} (mAU), micro-ultraviolet absorbance at 280 nm. Coomassie blue staining of the peak fractions following SDS–PAGE. Numbers on top of SDS-PAGE indicate elution volumes. Different frame color of SDS-PAGE corresponding to different protein in gel-filtration. Full blot of SDS-PAGE panels and full molecular weight ladder (M, kDa) are shown.

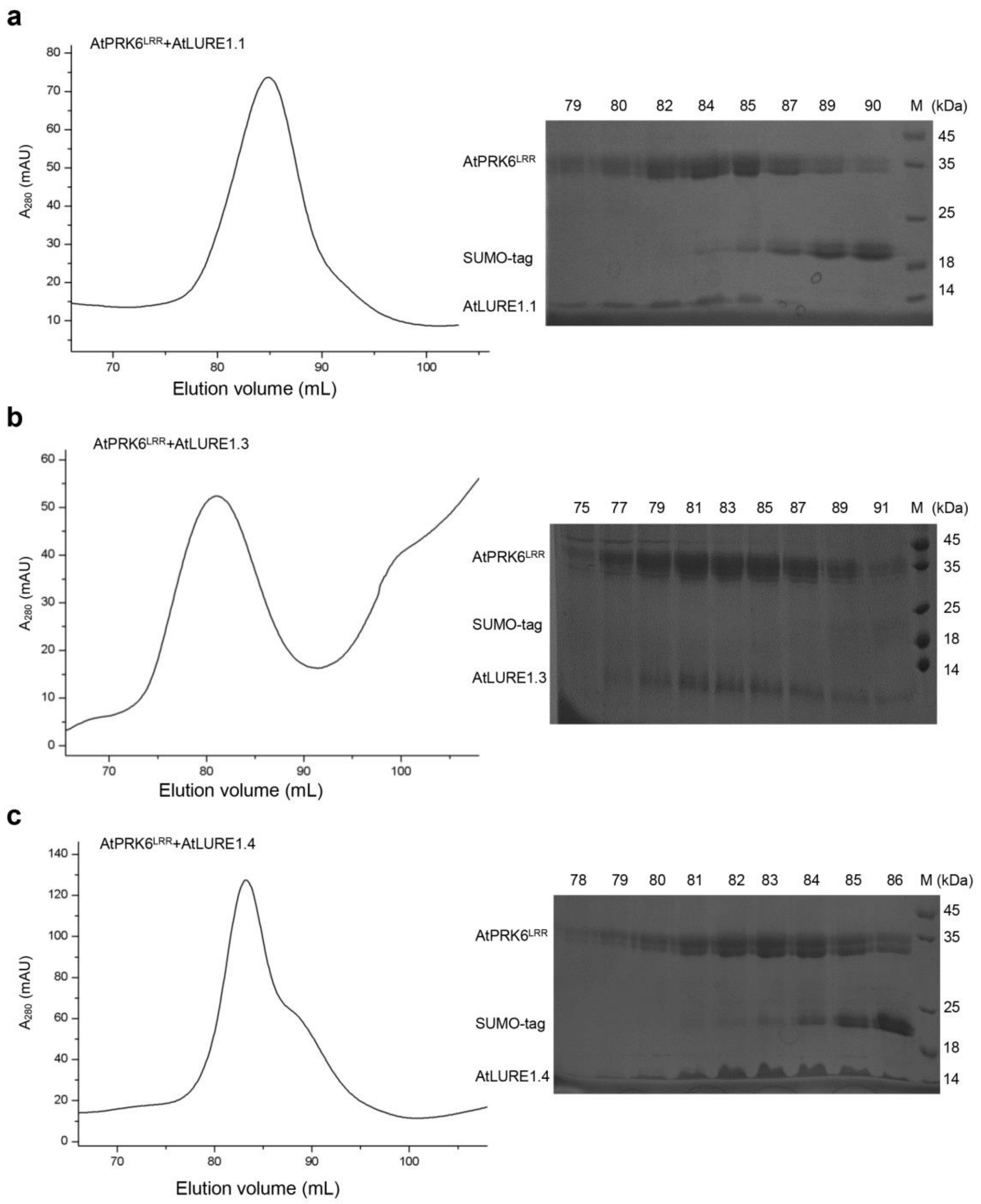

Supplementary Figure 2: AtPRK6^{LRR} form complexes with AtLURE1.1, AtLURE1.3 and AtLURE1.4 respectively under gel filtration

(a) Left panel: gel-filtration profile of AtPRK6^{LRR} and AtLURE1.1 co-expressed in insect cells. A_{280} (mAU), micro-ultraviolet absorbance at 280 nm. Right panel: coomassie blue staining of the peak fractions shown on the top following SDS–PAGE. The ~34kDa band shows the AtPRK6^{LRR}, the ~14kDa band shows the AtLURE1.1 peptide, they form a complex in gel filtration. The ~20kDa band represents the co-eluting residual SUMO protein in Ni-NTA purification. M, molecular weight ladder (kDa). Numbers on top of SDS-PAGE indicate elution volumes.

(b) Left panel: gel-filtration profile of AtPRK6^{LRR} and AtLURE1.3 co-expressed in insect cells. Right panel: coomassie blue staining of the peak fractions shown on the top following SDS–PAGE. The assays were performed as described in (a).

(c) Left panel: gel-filtration profile of AtPRK6^{LRR} and AtLURE1.4 co-expressed in insect cells. Right panel: coomassie blue staining of the peak fractions shown on the top following SDS–PAGE. The assays were performed as described in (a).

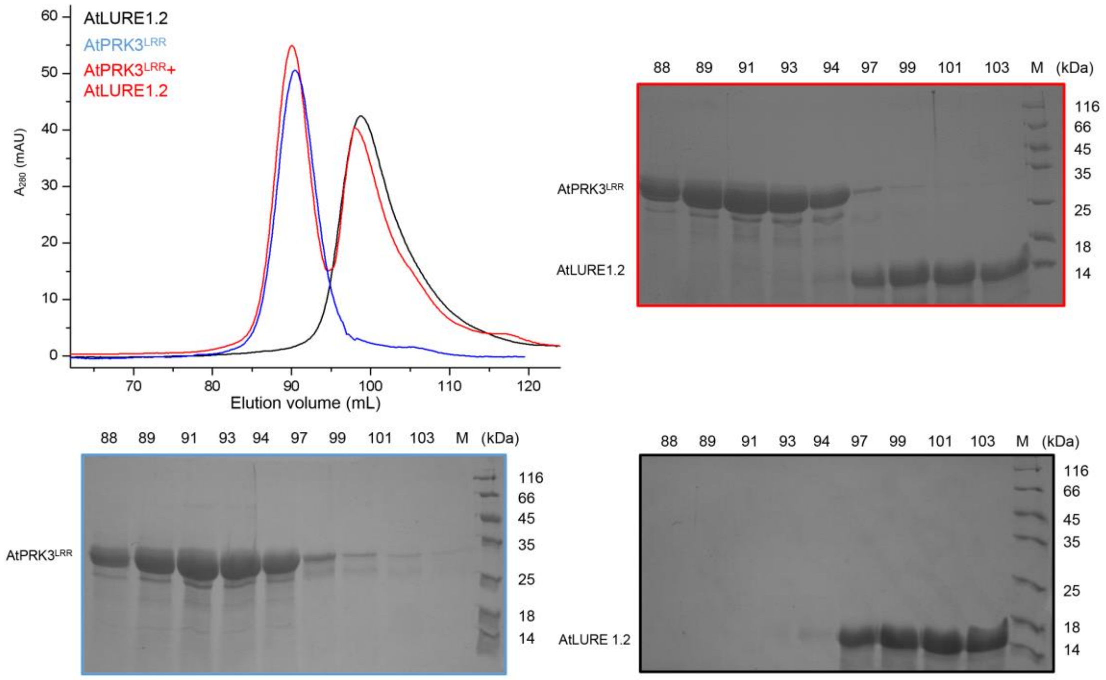

Supplementary Figure 3: No interaction to AtLURE1.2 was detected under gel filtration assay with AtPRK3^{LRR}.

Left panel: gel-filtration profile of AtPRK3^{LRR}, AtLURE1.2 and their mixture. A_{280} (mAU), micro-ultraviolet absorbance at 280 nm. Right panel: Coomassie blue staining of the peak fractions shown on the top following SDS–PAGE. M, molecular weight ladder (kDa). Numbers on top of SDS-PAGE indicate elution volumes. The assays were performed as described in Figure 1b.

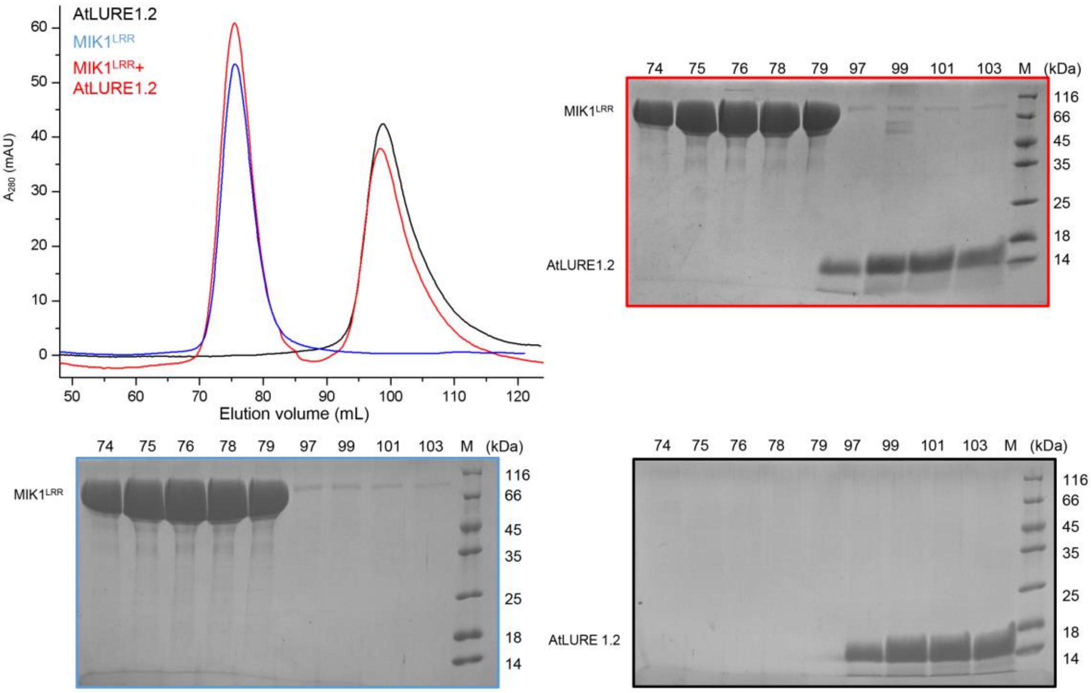

Supplementary Figure 4: No interaction to AtLURE1.2 was detected under gel filtration assay with MIK1^{LRR}.

Left panel: gel-filtration profile of MIK1^{LRR}, AtLURE1.2 and their mixture. A_{280} (mAU), micro-ultraviolet absorbance at 280 nm. Right panel: Coomassie blue staining of the peak fractions shown on the top following SDS–PAGE. M, molecular weight ladder (kDa). Numbers on top of SDS-PAGE indicate elution volumes. The assays were performed as described in Figure 1b.

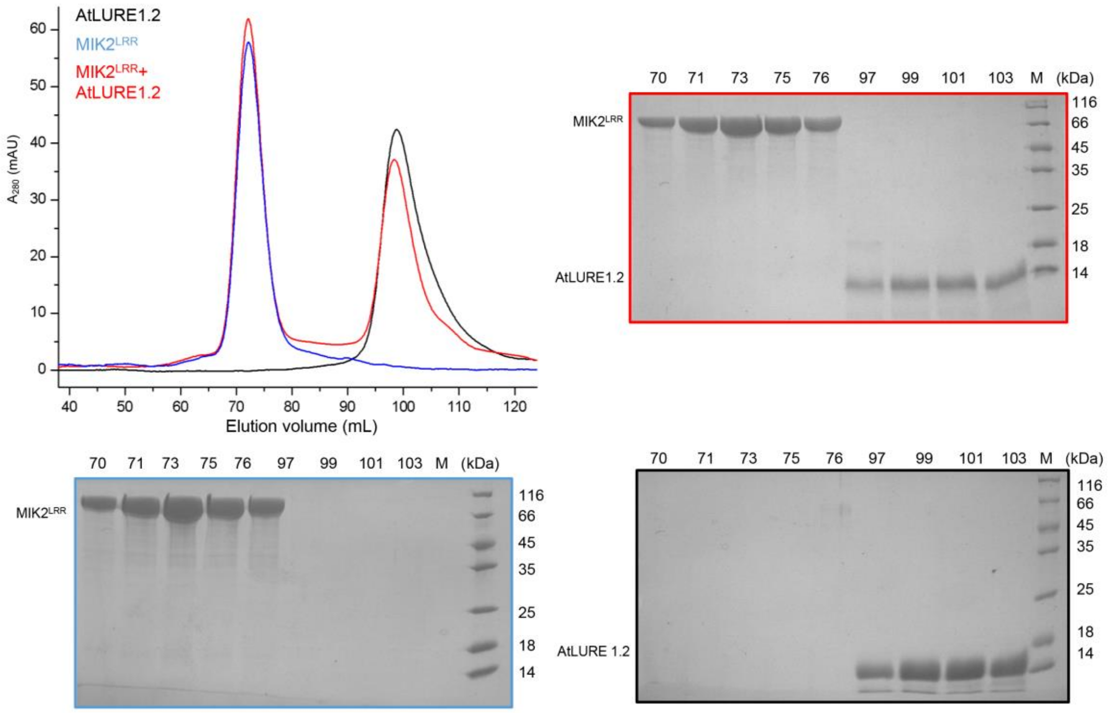

Supplementary Figure 5: No interaction to AtLURE1.2 was detected under gel filtration assay with MIK2^{LRR}.

Left panel: gel-filtration profile of MIK2^{LRR}, AtLURE1.2 and their mixture. A_{280} (mAU), micro-ultraviolet absorbance at 280 nm. Right panel: Coomassie blue staining of the peak fractions shown on the top following SDS–PAGE. M, molecular weight ladder (kDa). Numbers on top of SDS-PAGE indicate elution volumes. The assays were performed as described in Figure 1b.

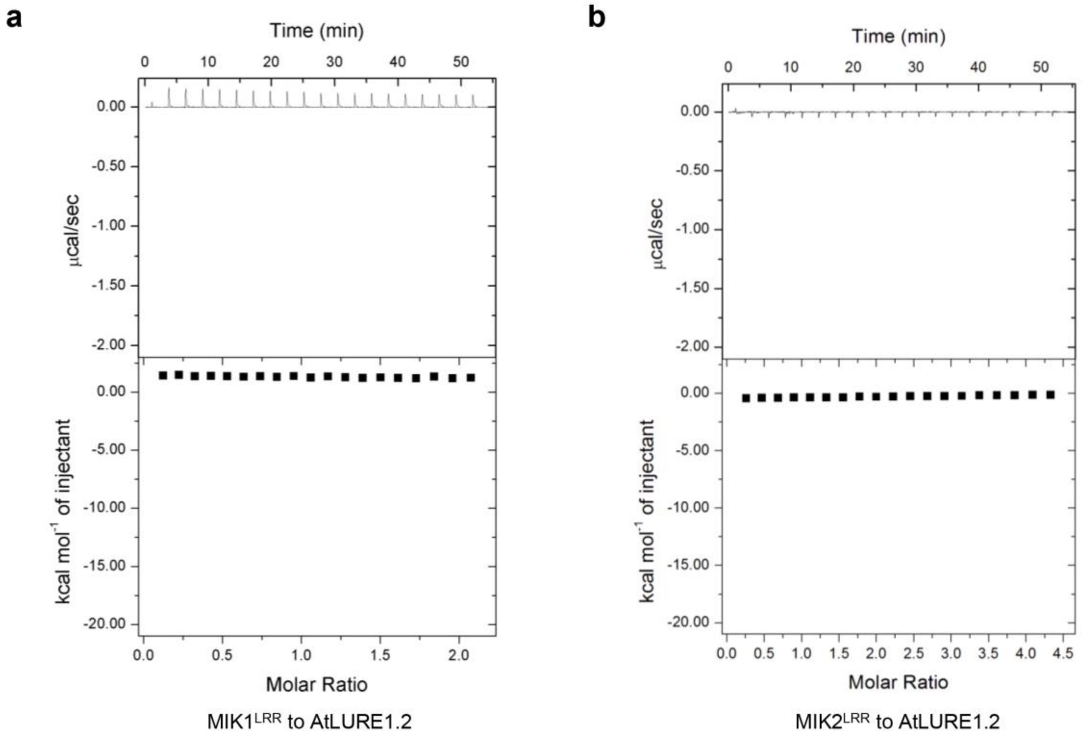

Supplementary Figure 6: Binding affinity between MIK1^{LRR}, MIK2^{LRR} and AtLURE1.2 was not detected in ITC assay.

(a) Upper panel: twenty injections of 0.5mM AtLURE1.2 solution were titrated into 0.05mM MIK1^{LRR} solution in the ITC cell. The area of each injection peak corresponds to the total heat released for that injection. Lower panel: the integrated heat is plotted against the molar ratio between AtLURE1.2 and MIK1^{LRR}.

(b) Binding affinity between MIK2^{LRR} and AtLURE1.2 by ITC. The assays were performed as described in (a).

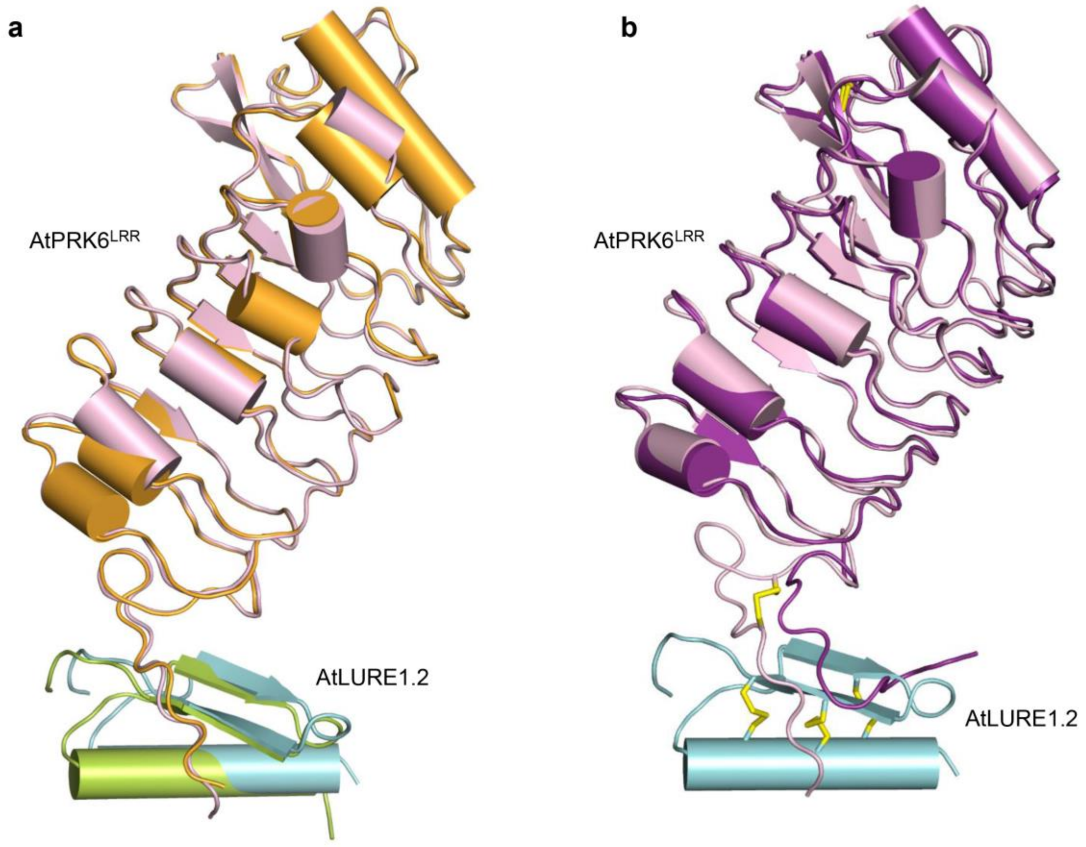

Supplementary Figure 7: Structural comparison of AtLURE1.2-AtPRK6^{LRR} complex and AtPRK6^{LRR} conformation in different states.

(a) Structural alignment of AtLURE1.2-AtPRK6^{LRR} protein complexes in two crystal forms. Structures of AtLURE1.2 and AtPRK6^{LRR} with higher resolution are presented in cyan and pink respectively, in green and orange with lower resolution, respectively.

(b) Structural alignment of the two AtPRK6^{LRR} molecules from one asymmetric unit of the higher resolution of the AtLURE1.2-AtPRK6^{LRR} complex structure. The structure of AtLURE1.2-free AtPRK6^{LRR} is shown in purple.

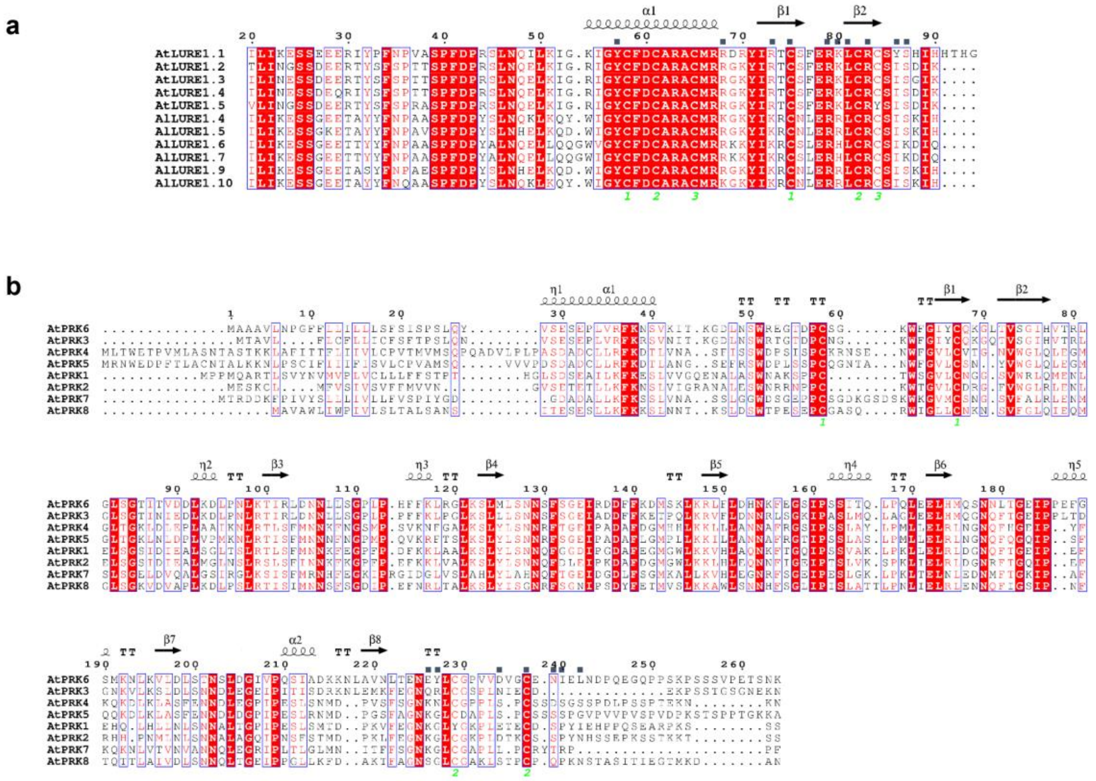

Supplementary Figure 8: Sequence alignment of AtPRKs and AtLUREs.

(a) Sequence alignment of LURE peptides in their mature forms from *A. thaliana* and *A. lyrata*. Conserved and similar residues are boxed with red ground and red font, respectively. Cysteine residues forming a disulfide bond are indicated by the same green numbers. The AtPRK6^{LRR} interacting amino acids of AtLURE1.2 are highlighted with blue squares on top. Most of the AtPRK6^{LRR}-interacting residues of AtLURE1.2 are highly conserved in members of LURE including *A. thaliana* and *A. lyrata*.

(b) Sequence alignment of the extodomains of AtPRK family members. The AtLURE1.2 interacting amino acids of AtPRK6^{LRR} are highlighted with blue squares on top. The AtLURE1.2-interacting residues of AtPRK6^{LRR} are not conserved in other AtPRK^{LRR} proteins.

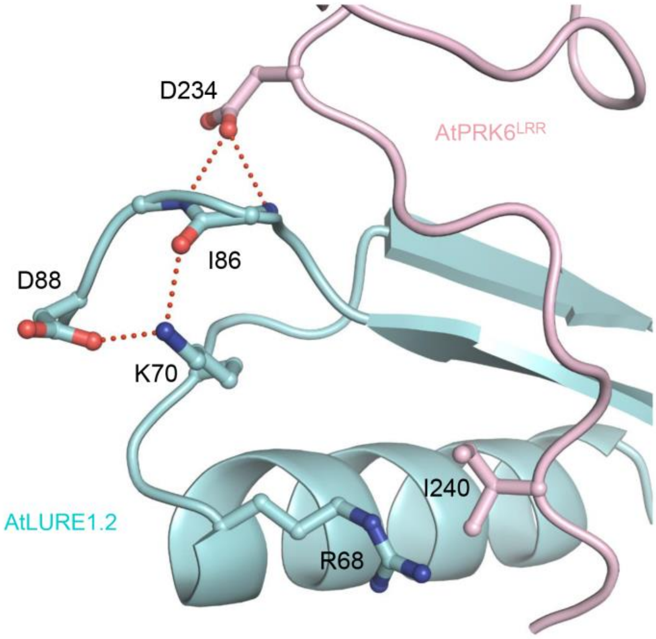

Supplementary Figure 9: Arg68 and Lys70 of AtLURE1.2 are involved in inter- and intra-molecular interaction, respectively.

Arg68 forms van der Waals contact with Ile240 of AtPRK6. Lys70 of AtLURE1.2 stabilizes the C-loop conformation of AtPRK6^{LRR}. Red lines indicate hydrogen bonds.

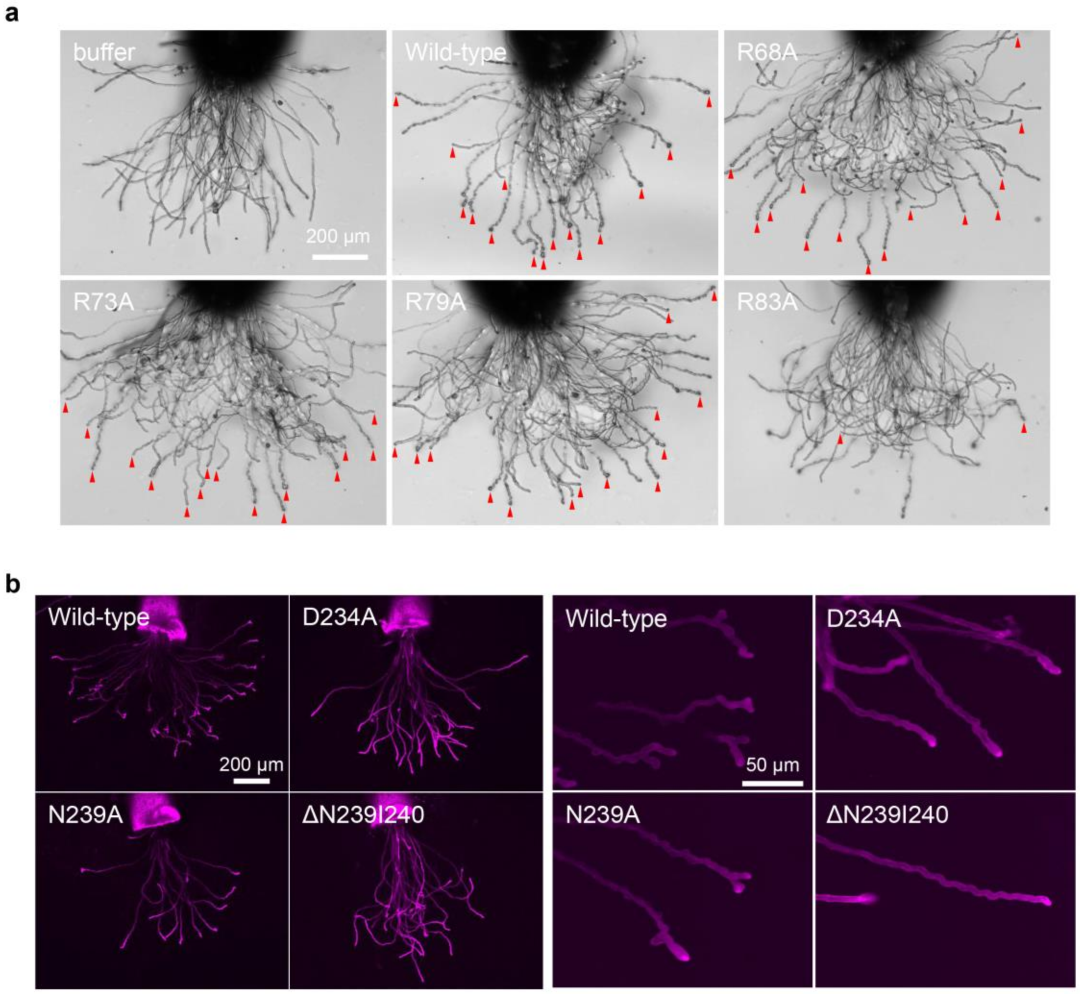

Supplementary Figure 10: Evaluation of reactivity of mutant LURE1.2 peptides and PRK6 receptors by wavy assay.

(a) *Semi-in-vivo* pollen tubes (wild type) were cultivated on the medium containing 250 nM AtLURE1.2 peptides of wild type and R68A, R78A, R79A, and R83A mutants, respectively. Pollen tubes show severe wavy behavior (red arrowheads) for AtLURE1.2 peptides with attraction activity. Note that wavy pollen tubes are decreased on R83A containing medium.

(b) *Semi-in-vivo* pollen tubes (prk6 transformed with PRK6 of wild-type, D234A, N239A, and ΔN239I240) were cultivated on the medium containing 250 nM wild-type AtLURE1.2 peptides. Pollen tubes with wild-type PRK6, as well as N239A, show severe wavy and branched behavior by sensing AtLURE1.2 peptides. Note that branched behavior is not observed in D234A and ΔN239I240 pollen tubes. Fluorescence is from mRuby2 fused to the C-terminal of PRK6 receptors, showing that all of mutant PRK6 receptors localize normally to the plasma membrane of the pollen tube tip.

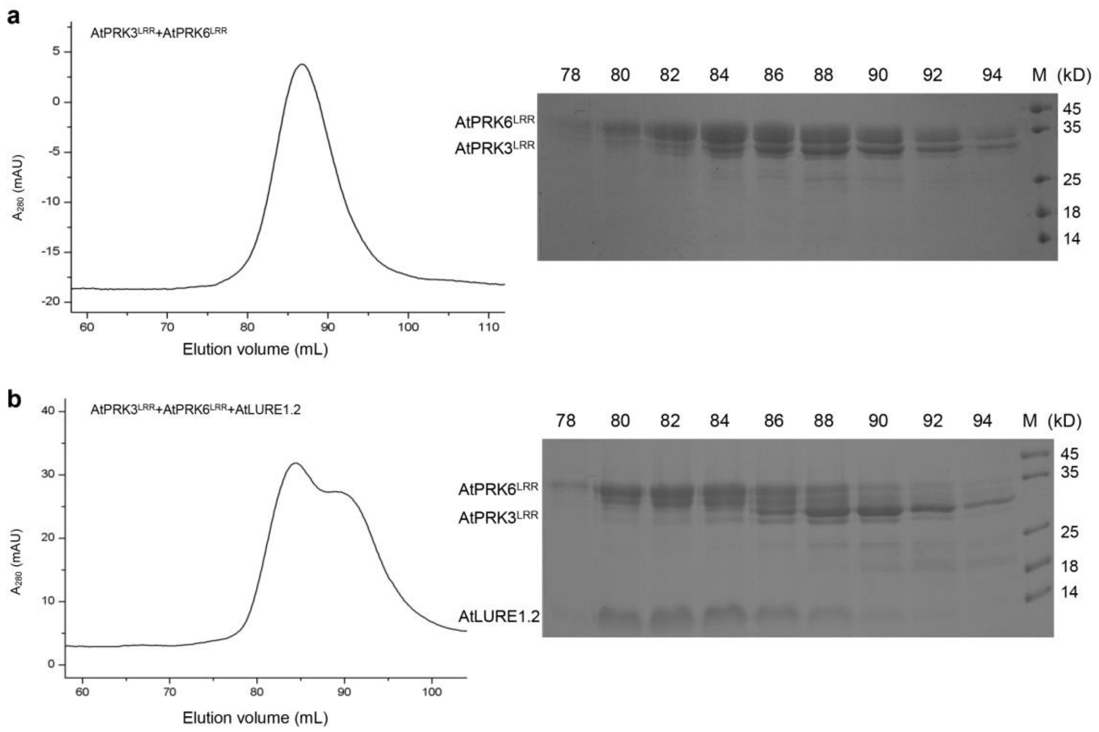

Supplementary Figure 11: AtPRK3^{LRR} shows no interaction with AtPRK6^{LRR} in the presence or absence of AtLURE1.2 *in vitro*.

(a) AtPRK3^{LRR} and AtPRK6^{LRR} do not interact with each other under gel filtration assay. Left panel: gel-filtration profile of the AtPRK3^{LRR} and AtPRK6^{LRR} proteins. A280 (mAU), micro-ultraviolet absorbance at 280 nm. Right panel: Coomassie blue staining of the peak fractions shown on the top following SDS–PAGE. M, molecular weight ladder (kDa). Numbers on top of SDS-PAGE indicate elution volumes.

(b) AtPRK3^{LRR} and AtPRK6^{LRR} do not form a complex in the presence of AtLURE1.2 under gel filtration assay. The assays were performed as described in (a).
